# Effects of Different Thickness Combinations of Core and Veneer Ceramics on Optical Properties of CAD-CAM Glass-Ceramics

**DOI:** 10.1155/2019/5856482

**Published:** 2019-03-04

**Authors:** Wol Kang, Jong-Kyoung Park, Woong-Chul Kim, Hae-Young Kim, Ji-Hwan Kim

**Affiliations:** ^1^Department of Dental Laboratory Science and Engineering, College of Health Science, Korea University, Seoul, Republic of Korea; ^2^Institute of Health Science Research & Department of Dental Laboratory Science and Engineering, College of Health Science, Korea University, Seoul, Republic of Korea

## Abstract

The goal of this study was to evaluate the effects of different core and veneer thicknesses on the translucency (T%), average light transmittance (T), translucency parameter (TP), contrast ratio (CR), and spectral reflectance (R) of glass-ceramics using a computer-aided design/computer-aided manufacturing (CAD-CAM) process. In all, 42 specimens (11mm × 11mm) were prepared and divided into six groups (*n* = 7 for each group). Core materials (IPS e.max CAD; IPS Empress CAD, LT A2 shade) of different thicknesses (0.8, 1.0, and 1.2mm) were fabricated. Each veneer material (thicknesses of 0.7, 0.5, and 0.3mm) was combined with its compatible core ceramic. Each core material was overlapped with its corresponding veneer material to obtain a 1.5-mm thickness by using three different combinations: (0.8+0.7), (1.0+0.5), and (1.2+0.3) mm. A spectrophotometer and color data software were used to measure the T%, T, TP, CR, and R values of each ceramic. The results were statistically analyzed using two-way analysis of variables (ANOVA) and regression analysis (*p*<0.05). Two-way ANOVA revealed that T%, T, TP, and CR were significantly influenced by the different thicknesses of the core-veneer combinations (*p*<0.001). At a certain thickness, as the veneer thickness increased and core thickness decreased, T and T% all increased. Regression analysis of the ceramic materials indicated a reduction in T and T% for certain core-veneer combinations. Analysis also revealed that T% and T were all affected by different core-veneer combinations. The T% value was 74.31 for the EM group and 72.81 for the EP group when the thickness of the core was 1.2 mm and the veneer was 0.3 mm. The R value of EM2 was lower than EM1 and EM3. In conclusion, the optical properties were influenced by different core-veneer combinations.

## 1. Introduction

In recent years, increased demand for esthetic dentistry has contributed to the increased popularity of ceramic restorations [1]. Because ceramic restorations do not have a metal substructure, their light transmission and scattering properties are similar to those of natural teeth [2]. In particular, ceramics fabricated using computer-aided design/computer-aided manufacturing (CAD-CAM) processes have become popular in dentistry [3] based on several advantageous features. These features include the adequate restoration strength provided by ceramics and natural appearance of such restorations. Furthermore, ceramic restorations are easier to use and the restoration process is faster and more accurate [4].

Zirconia is a popular ceramic material used in CAD-CAM processes. However, it has been reported that zirconia tends to fracture based on residual thermal stresses within the material [5]. Additionally, the translucency of zirconia is inferior to that of CAD-CAM glass-ceramics [6]. Glass-ceramics show relatively better ability to bond with resin cement, so that they are mainly applied for reconstructing anterior teeth such as crowns and laminates [2, 7]. The use of lithium disilicate CAD-CAM glass-ceramics has increased significantly because of superior esthetics [6, 8], relatively high flexural strength [9], and ability to bond with etched dentin and enamel [10]. Lithium disilicate glass-ceramics have higher strengths than leucite-reinforced glass-ceramics [9], but these strengths do not ensure better clinical performance [11]. Therefore, clinicians tend to prefer CAD-CAM glass-ceramics [11].

Previous studies have indicated that the core ceramic controls the Munsell value of a restoration and the veneer ceramic can be used to match the internal color [12]. These characteristics mimic a real tooth's vitality and shade by recreating an appropriate mixture of scattering and light absorption [12]. Therefore, ceramic systems require core-veneer combinations to imitate a natural tooth [13].

Translucency is one of the most important factors in controlling esthetic outcomes [2]. Appropriate translucency can make a ceramic appear more natural [14]. Translucency may be determined by the transmitted light through the object or the reflected light (R) of the object [15]. Therefore, light is an important factor in evaluating the translucency of a substrate [12]. There are several methods for evaluating translucency, including light transmittance (T%) [11], contrast ratio (CR) [14], and translucency parameter (TP). Of these methods, TP and CR have been frequently used to measure the translucency of ceramics [6, 16–18]. Recently, the use of T% has increased [11, 12, 19]. Therefore, this study used R, TP, CR, and T% as standard measures.

To create successful clinical restorations, accurate translucency must be accompanied by accurate color [19]. Previous studies have studied the color of cores and veneers of different thicknesses, but studies of translucency have not been conducted separately [20]. Based on the importance placed on esthetics in ceramic restorations, many studies have investigated various parameters, such as different types of materials, their thicknesses, fabrication techniques, and illuminants, which can affect optical properties [6, 16–18]. Although several reports have described the effects of thickness on optical properties, there is an insufficient number of studies addressing the optical properties of CAD-CAM glass-ceramics with different core-veneer combinations.

In this study, we focused on this aspect of ceramic analysis and investigated the T%, T, CR, TP, and R of different CAD-CAM glass-ceramic core-veneer combinations. The null hypothesis was that optical properties would not be influenced by different core-veneer combinations of CAD-CAM glass-ceramics.

## 2. Materials and Methods

In this study, lithium disilicate (IPS e.max CAD, LT A2 shade, Ivoclar Vivadent, Schaan, Liechtenstein) and leucite-reinforced (IPS Empress CAD, LT A2 shade, Ivoclar Vivadent, Schaan, Liechtenstein) ceramics were tested. Each veneer material was paired with a compatible core ceramic (see Table 1).

A total of 42 specimens (11mm × 11mm) were fabricated for analysis. Cores of different thicknesses (0.8mm, 1.0mm, and 1.2mm) were fabricated and each group included seven specimens. Veneers of different thicknesses (0.7mm, 0.5mm, and 0.3mm) were also fabricated and divided into groups of seven specimens. The group names are based on the name of the ceramic core (IPS e.max CAD:EM, IPS Empress CAD:EP) and the associated numbers are determined by the combined thickness of the core and the veneer: 1 = (0.8 + 0.7), 2 = (1.0 + 0.5), and 3 = (1.2 + 0.3). The size of the specimens was determined using power analysis (actual power = 90%, *α* = 0.05).

Each of the core samples was cut with consideration for shrinkage and the amount of material removed during cutting (Techcut 5™ Precision High-Speed Saw, Allied High Tech Products Inc., Compton, USA). The cut specimens were fired according to manufacturer instructions. Each specimen was fabricated in the shape of a square by using a diamond cutter (Wafering Blades: Metal-Bonded Blades, Allied High Tech Products Inc., Compton, USA). The veneers were fabricated in a silicone mold and were designed with consideration for firing shrinkage, deformation, and the amount of material removed during cutting. The veneer specimens were also fired according to manufacturer instructions. Subsequently, the surfaces of the specimens were ground and polished by a horizontal-spindle surface-grinding machine (HRG-150, AM Technology, Chungcheongnam-do, South Korea) using silicon carbide abrasive papers (SPL-15 Grind X, Okamoto Co., Yokohama, Japan) with grits of 200, 400, 600, 800, 1200, and 1500. All specimens were cleaned in an ultrasonic cleaner (SD Ultrasonic Cleaner, Mujigae, Daejeon, South Korea) for 10 min using distilled water.

Each core sample was overlapped with its corresponding veneer sample to obtain a combined thickness of 1.5mm using different combinations of core and veneer thicknesses. The combinations were labelled as follows: (0.8+0.7) mm = 1, (1.0+0.5) mm = 2, and (1.2+0.3) mm = 3. The lithium disilicate and leucite-reinforced ceramics both have refractive indices of approximately 1.5 [21]. A drop of optical fluid (1.5 index of refraction fluid, Cargille Lab, Cedar Grove, USA) was applied between the overlapping specimens to ensure good optical contact and minimize scattering at the interface [18]. The thicknesses of the specimens were measured at three different points using a digital micrometer (Quantu Mike Coolant-Proof Micrometer, Mitutoyo, Kanagawa, Japan).

The optical properties of the specimens were measured using a dual-beam spectrophotometer (CM-3600A Spectrophotometer, Konica Minolta, Tokyo, Japan). Prior to measurement, the spectrophotometer was calibrated using a zero-transmittance calibration plate, which was then removed. The optical properties were measured in the transmittance chamber of the spectrophotometer. Spectral transmittance and reflectance data were collected at 10nm intervals of 400–700 nm using the International Commission on Illumination (CIE) standard illuminant D65. The conditions for illuminating and viewing were determined based on the CIE diffuse/8°. The measurement area was 4mm in diameter. A customized sample holder was installed in the transmittance chamber to hold the samples in place during measurement. To increase the reliability of measurement at the same position, a ledge was fabricated using putty (Blu Tack, Bostik, Borgholzhausen, Germany) by displaying three points on the measurement aperture where the edge of the specimen was located.

To measure T%, the luminance without a specimen (Luminance_source_) was measured and used as a baseline value. Subsequently, the luminance with a specimen (Luminance_specimen_) was calculated for each specimen using color data software (Spectra Magic NX CM-S100w, Konica Minolta, Tokyo, Japan). T% was then calculated using the following equation:

(1) $$\begin{array}{c} T\%=\left(\;\frac{{Luminance}_{specimen}}{{Luminance}_{source}}\;\right)\times 100 \end{array}$$

To calculate the average light transmittance (T), the sum of transmittances at each wavelength was divided by the number of data points (31).

To calculate the average spectral reflectance (R), the sum of reflectance at each wavelength was divided by the number of data points (31). Where reflectance measurements were carried out, the specimens were placed on a black (R_b_) and white (R_w_) backing.

The relationship between the thickness, T%, and T values of different core-veneer combinations was analyzed via regression analysis based on the following exponential function:

(2) $$\begin{array}{c} y=a\cdot \exp\left(\;bx\;\right), \end{array}$$

where* y* is the observed T or T% value,* x* is the thickness of the sample, and* a* and* b* are constants.

Translucency parameter (TP) of the specimens was calculated using the following equation:

(3) $$\begin{array}{c} TP={\left[\;{\left(\;L_{W}^{*}-L_{B}^{*}\;\right)}^{2}+{\left(\;a_{W}^{*}-a_{B}^{*}\;\right)}^{2}+{\left(\;b_{W}^{*}-b_{B}^{*}\;\right)}^{2}\;\right]}^{1/2} \end{array}$$

where *L*^*∗*^ represents the degree of lightness, *a*^*∗*^ represents the degree of redness/greenness, *b*^*∗*^ represents the degree of yellowness/blueness, *W* represents that the specimens were placed on a white background, and *B* represents that the specimens were placed on a black background [22].

Contrast ratio (CR) of the specimens was calculated using the following equation:

(4) $$\begin{array}{c} \frac{Y_{b}}{Y_{w}} \end{array}$$

the variable,* Y*, refers to the spectral reflectance of the specimens.

An independent-sample* t*-test was performed to compare specimen thicknesses. Two-way analysis of variables (ANOVA) was used to identify the effects of different core-veneer combinations and types of ceramic materials (*p*< 0.05). A Tukey multiple comparison test was performed to identify significant group differences (*α*=0.05). All statistical tests were performed using SPSS version 22.0 (IBM Corporation, Armonk, NY, USA).

## 3. Results

The average thicknesses and standard deviations (SDs) are shown in Table 2. The* p*-values of the groups are as follows: EM1 and EP1 (*p*=0.465); EM2 and EP2 (*p*=0.931); EM3 and EP3 (*p*=0.904). The independent-sample* t*-test revealed no significant differences between the thicknesses of the ceramic materials (*p*>0.05).

The mean values of T%, T, TP, CR, R_w_, and R_b_ for the different core-veneer combinations investigated in this study are listed in Table 3. Two-way ANOVA revealed statistically significant differences between the effects of materials with different core-veneer combinations on T%,T, TP, CR, R_w_, and R_b_ (*p<*0.05) (Table 3).

Regression analysis was performed to evaluate the association between T and different core-veneer combinations. The calculated regression equations and correlation coefficients (R^2^) for the lithium disilicate and leucite-reinforced ceramic materials are as follows:

(5) T=28.69exp⁡−1.989x;  R2=0.771,  for  EMT=33.32exp⁡−3.517x;  R2=0.909,  for  EP

Similarly, regression analysis was performed to evaluate the association between T% and the different core-veneer combinations. The calculated regression equations and correlation coefficients (R^2^) for both ceramic materials are as follows:

(6) T%=76.92exp⁡–2.345x;  R2=0.751,  for  EMT%=80.37exp⁡–4.091x;  R2=0.829,  for  EP

## 4. Discussion

The results of this study highlight the differences in T%, T, TP, CR, and R of different core-veneer combinations and different types of CAD-CAM glass-ceramics. The null hypothesis, that T%, T, TP, CR, and R would not be influenced by different core-veneer combinations, was rejected.

The edge-loss phenomenon is one of the most important factors in translucency studies [14]. A previous study indicated that the observation port for translucency analysis should be two or three times larger than the size of the light beam to weaken the edge-loss phenomenon [23]. Therefore, the present study, a spectrophotometer with a measurement area of 4mm, was used to measure 11mm specimens.

In several studies [6, 16, 24, 25], A2 shade was used to measure the optical properties values. Therefore the present study used A2 shade. However, because the method and material are different, it is not possible to compare it with other studies. Although some studies have reported on the optical properties of thickness [6, 11, 24, 26], none of these studies addressed such effects on CAD-CAM glass-ceramics of different core and veneer combinations. Therefore, the present study used CAD-CAM glass-ceramic to examine the optical properties of different core and veneer combinations.

To provide a natural appearance, ceramic systems (i.e., different core-veneer combinations) should exhibit variable translucency [13]. The thickness of a ceramic significantly affects its translucency and light transmittance [16, 23, 26, 27]. It is generally recommended that a ceramic restoration should be 1.5 mm thick [28]. Additionally, Heffernan et al. [16] suggested that the core should be at least 0.8 mm thick when combined with different veneers. Therefore, the thickness of the core-veneer combinations analyzed in this study was set to 1.5 mm. The thicknesses of the cores were set to 0.8, 1.0, and 1.2 mm, which are thicker than the manufacturer-recommended minimum core thickness of 0.7 mm. The thicknesses of the veneers were set to 0.7, 0.5, and 0.3 mm. These values were chosen because the veneer should be thinner than the core. This study showed that the optical properties changed with the thicknesses of the core and veneer. Therefore, to increase the reliability of thickness measurements, the thickness of each core and veneer was measured three times, followed by an independent sample* t*-test. The results revealed no statistically significant thickness differences.

As the veneer thickness increased and core thickness decreased, T, T%, and TP increased and CR decreased (Table 3). These results suggest that both ceramic materials are dependent on the core and veneer thickness. Heffernan et al. [16] reported that the translucency of core materials increases after they are veneered and glazed. In this study, we polished the specimens using silicon carbide abrasive papers (1500 grit) but did not subject them to glazing. However, Saracet al. [29] reported that polished ceramics exhibit a surface smoothness similar to that of glazed ceramics. This implies a strong correlation between our results and previously reported results. However, as the veneer thickness increased and the core thickness decreased, the R of the EP group decreased but the EM group did not. This characteristic may be related to the microstructures of the ceramics. Because lithium disilicate is opaque with small interlocking needle-like crystals, it appears to be less influenced by layering. However, leucite-reinforced ceramics are considered to be affected by layering because they are less dense and undergo single-crystal formation with no interlocking between crystals [30].

T% was significantly affected by different core-veneer combinations in the EM and EP groups (Table 3). This result is consistent with that reported in a previous study, where it was shown that different core-veneer combinations have an impact on the T% value of a restoration [17]. Additionally, in this study, the rate of increase in T% of the EP group was higher than that of the EM group (Table 3). This is consistent with results reported in a previous study [30]. It is particularly noteworthy that when the core was 1 mm thick and the veneer was 0.5 mm thick, the T% values of the two materials were similar. This is similar to the results of previous studies in which IPS e.max CAD and IPS Empress CAD ceramics demonstrated similar T% values with core thicknesses of 1 mm [25]. However, when the core was 1.2 mm thick and veneer was 0.3 mm thick, the T% value was higher for the EM group than for the EP group. When the core was 0.8 mm thick and veneer was 0.7 mm thick, the T% value was lower for the EM group than for the EP group. This is different from previous studies that lithium disilicate is more opaque at lower wavelengths than leucite-reinforced glass-ceramics. This difference may be a result of the relative refractive index. In ceramic systems consisting of particles and a medium, an important factor in the scattering of light is the relative refractive index between the particles and medium [31]. Lithium disilicate and leucite-reinforced ceramics have refractive indices of 1.55 and 1.51, respectively [21]. When the refractive index constant between the indices of the high-index material and low-index material exceeds 1.1, an object can emit brilliant opalescent colors [32]. For this reason, the various core-veneer combinations examined in this study may have yielded different results. Therefore, CAD-CAM glass-ceramics are considered to be affected by different core-veneer combinations.

When examining the effects of T, materials gradually lose their strength as they pass through the light beam. This phenomenon is caused by the way light interacts with a material, causing scattering or absorption. According to the regression analysis of the exponential function of T, the larger the value of* b*, the greater the degree of light scattering and/or absorption [33]. The absolute value of* b* for the EM group was 1.989, whereas that for the EP group was 3.517. Because the EP group has a larger absolute value of* b*, the degree of scattering and/or absorption of light is considered to be greater than that for the EM group.

This study did not completely simulate a clinical situation because the specimens were subjected to various analysis techniques rather than used directly as ceramic restorations. Furthermore the core ceramic and the veneer ceramic specimens were overlapped rather than bonded. It is expected to have an impact on the transmittance. However, limitation of present study controls the edge loss. For this purpose, optical fluid (refractive index 1.5), which has been reported to provide good optical contact [18], was used in the present study to decrease any edge loss. Therefore, future clinical studies should thoroughly assess that the optical properties of the core-veneer combination are bonded.

## 5. Conclusions

Within the limitations of this study, the following conclusions were drawn. The T%, T, TP, CR, and R of ceramic core-veneer combinations are affected by the thicknesses of individual components even though the overall thickness is the same.

To analyze the T%, T, TP, CR, and R of ceramic restorations, it should be considered to have information regarding different core-veneer combinations. Therefore, even for a consistent total thickness, it is important to have an understanding of the optical properties of different core-veneer combinations in ceramic restorations. Clinicians may use this knowledge regarding different core-veneer combinations to better match a given type of restoration to a natural tooth.

## Figures and Tables

**Table 1:** Material types and properties.

Group^a^	Material		Type	Color	Manufacturer
EM	Core	IPS e.max CAD	Lithium disilicate glass ceramic	LT A2	IvoclarVivadent
	Veneer	IPS e.max Ceram	Nano-fluorapatite glass-ceramic	TI 1	IvoclarVivadent
EP	Core	IPS Empress CAD	Leucite-reinforced glass ceramic	A2	IvoclarVivadent
	Veneer	IPS Empress Esthetic Veneer	Feldspathic porcelain	T neutral	IvoclarVivadent

^a^EM:lithium disilicate glass-ceramic, EP: leucite-reinforced glass-ceramic

**Table 2:** Average thicknesses of specimens of various specimen assemblies with different core-veneer combinations.

Group^A^	Core + Veneer	Mean	SD
EM 1	0.8 + 0.7	1.502	0.001
EM 2	1.0 + 0.5	1.511	0.002
EM 3	1.2 + 0.3	1.503	0.001
EP 1	0.8 + 0.7	1.504	0.001
EP 2	1.0 + 0.5	1.504	0.002
EP 3	1.2 + 0.3	1.508	0.002

^A^EM: lithium disilicate glass-ceramic, EP: leucite-reinforced glass-ceramic

**Table 3:** Mean T, T%, TP, CR, R_w_ and R_b_ values of various specimen assemblies with different core-veneer combinations.

Group^A^			Mean(SD)			
	T	T%	TP	CR	R_W_	R_B_
EM 1	26.49 (0.79)^a^	74.31 (0.88)^ac^	2.41 (0.025)^a^	0.64 (0.013)^ab^	51.87 (0.47)^a^	33.26 (0.84)^a^
EM 2	25.12 (0.92)^a^	72.76 (1.17)^a^	2.39 (0.013)^ab^	0.65 (0.004)^b^	50.80 (1.19)^a^	33.18 (0.61)^a^
EM 3	22.51 (0.97)^b^	69.61 (1.25)^b^	2.37 (0.034)^b^	0.66 (0.013)^c^	50.86 (0.78)^a^	34.04 (0.82)^a^
EP 1	29.64 (0.45)^c^	75.97 (0.47)^c^	2.47 (0.005)^c^	0.62 (0.002)^d^	59.22 (0.59)^b^	36.55 (0.37)^b^
EP 2	26.60 (1.11)^a^	72.81 (1.30)^a^	2.45 (0.009)^c^	0.64 (0.004)^a^	59.41 (0.60)^b^	37.77 (0.40)^c^
EP 3	22.60 (1.11)^b^	67.79 (2.33)^b^	2.40 (0.011)^a^	0.67 (0.005)^c^	59.64 (0.31)^b^	39.87 (0.44)^d^

P (material)	<.001	0.941	<.001	<.001	<.001	<.001
P (thickness)	<.001	<.001	<.001	<.001	0.321	<.001
P (mat.thick.)	<.001	<.05	0.156	<.05	<.05	<.001

^A^EM: lithium disilicate glass-ceramic, EP: leucite-reinforced glass-ceramic

^B^Values with superscripts indicate statistically significant differences (*p *> 0.05) according to the post hoc test (Tukey multiple comparison test was used for statistical analysis).

^C^P-values were obtained from two-way ANOVA.

## Data Availability

The [EXCEL TYPE] data used to support the findings of this study are available from the corresponding author upon request.
